# Characterization of fecal bacterial microbiomes according to fecal color, consistency, and sample type in piglets before and after weaning

**DOI:** 10.3389/fvets.2026.1815748

**Published:** 2026-05-22

**Authors:** C. Vaggi, J. C. Vötterl, F. Lerch, F. Yosi, S. Koger, S. Ricci, D. Verhovsek, B. U. Metzler-Zebeli

**Affiliations:** 1 Centre for Animal Nutrition and Welfare, Clinical Department for Farm Animals and Food System Transformation, University of Veterinary Medicine Vienna, Vienna, Austria; 2 Christian-Doppler Laboratory for Innovative Gut Health Concepts of Livestock, University of Veterinary Medicine Vienna, Vienna, Austria; 3 Department of Animal Science, Faculty of Agriculture, University of Sriwijaya, Palembang, South Sumatra, Indonesia; 4 Clinical Centre for Population Medicine in Fish, Pig and Poultry, Clinical Department for Farm Animals and Food System Transformation, University of Veterinary Medicine Vienna, Vienna, Austria

**Keywords:** bacterial abundances, fecal phenotype, fecal sampling, gut microbiome, intestinal development, weaning piglets

## Abstract

Fecal samples are widely used as a proxy for the large intestinal microbiota; however, phenotypic characteristics (e.g., color and consistency) may be associated with divergent microbial profiles, especially around weaning, when diet and physiological adaptation rapidly alter gut function. The relationship between fecal phenotype, sample type, and piglet gut microbiota under physiological conditions remains poorly understood. This study investigated the bacterial communities in different fecal phenotypes of piglets shortly before and immediately after weaning. The fecal consistency of 192 piglets across two replicate batches was scored daily from day of life (DoL) 28 to 36, and fecal or rectal swab samples were collected at DoL28 and DoL33. The samples were classified by type (feces/swab), color (brown/yellow), and consistency (balls/liquid). DNA was extracted for quantification of total bacterial gene copies and 16S rRNA gene sequencing, and microbial composition was analyzed using Quantitative Insights Into Microbial Ecology 2 (QIIME2), Statistical Analysis System (SAS), and R. Fecal consistency changed markedly over time, shifting from predominantly ball-shaped on DoL28-32 to softer feces thereafter (*p* < 0.001). Age strongly influenced microbiota structure, with marked increases in relative abundance of *Prevotella* and *Alloprevotella* from DoL28 to DoL33, whereas the abundance of *Escherichia, Methanobrevibacter,* and *Fusobacterium* declined. Microbial communities differed between sample types, with swabs potentially reflecting mucosa-associated taxa more closely than fecal samples. Shannon and Simpson indices indicated reduced diversity in yellow and liquid feces on DoL28 (*p* < 0.001). Swabs and yellow liquid feces on DoL28 showed higher relative abundances of *Escherichia*, *Bacteroides*, and *Fusobacterium*, whereas brown ball-shaped feces were enriched in *Lachnospiraceae*, *Prevotella*, and *Lactobacillus* on both sampling days (*p* < 0.05). Overall, each fecal phenotype exhibited a distinct bacterial signature, and the sample type influenced the composition of the captured community. Monitoring fecal phenotypes alongside selecting appropriate sample types may enhance the interpretation of microbiome data and offer a practical, non-invasive approach to assess gut health during the critical weaning period.

## 1 Introduction

The immediate postweaning period is a major time point in the health, welfare, and development of young pigs (1). Numerous studies have focused on the fecal microbiota of piglets to understand the dynamics of the gut microbiota during the weaning transition, as feces are easy to collect and allow multiple samplings (2–4). The abrupt removal of sow milk at weaning leads to drastic alterations in substrate availability for gut microbial taxa, resulting in a large decline in taxa related to the fermentation of milk glycans, such as *Lactobacillaceae* and *Bacteroidaceae* (2, 5). They are replaced by taxa that are more capable of degrading plant starches and fibers, such as *Prevotellaceae, Lachnospiraceae*, and the yeast fungus *Kazachstania* (2, 5). These microbial changes can lead to decreased immune tolerance of the host animal to its own microbiota, which can make the piglet more susceptible to gut disturbances (2, 5). A previous study showed that analysis of the microbial composition in feces may be useful to discriminate piglets susceptible to postweaning diarrhea from healthy piglets 1 week after birth (6). Therefore, the analysis of the microbial composition in feces early in the life of piglets may be a helpful tool for early diagnosis and timely intervention to prevent gut dysbiosis (3, 6, 7). Regarding the practical approach, reliable analysis of fecal microbiota requires the use of freshly excreted feces, which might not always be collectible in younger pigs (2, 4), followed by costly DNA extraction, quantitative polymerase chain reaction (qPCR), and sequencing, which may limit its applicability in certain settings. If fresh feces cannot be obtained, alternative rectal swabs can be used (2). Cotton rectal swabs resist environmental contamination (e.g., soil and urine), thus preserving a more reliable microbial signature for further analysis (4, 8). Controversial findings exist in the literature regarding whether rectal swab samples are representative of fresh feces (2, 4, 8, 9). Owing to their proximity to the mucosal surface, rectal samples comprise more glycoprotein-degrading taxa, such as *Clostridiaceae*, *Campylobacteriaceae*, and *Veillonellaceae*, than fecal samples (2). However, other studies have proposed that rectal swabs can represent a suitable alternative to fecal samples, concluding that differences in their microbiota profiles are relatively small and a consequence of greater inter-individual and dietary variation than sample-type variability (4, 8). Overall, the phenotype of the feces of diarrhetic piglets is characteristic, owing to its liquid form and distinguishing smell. Generally, the fecal consistency of piglets varies from hard pellets to liquid, whereas fecal color varies from yellowish to gray to dark brown (2). Owing to the peculiarities of the weaning period (i.e., social and dietary stress and immature gut functions) (1), it is common for piglets to present with softer, yellowish to light brown feces immediately after weaning (7). Color changes and reduced consistency of feces in healthy piglets may be associated with feed components, bile secretion, and/or osmotically active microbial metabolites due to increased microbial activity on undigested feed residues in the gut lumen, rather than being exclusively associated with increased expression of virulence factors in the pathobionts (2). Therefore, assessing feces based on their phenotype may be a cost-effective and rapid method for determining abnormal gut homeostatic conditions. However, there is still little information available on whether the feces of a certain phenotype comprise similar indicator bacteria around weaning. Previous results from a small-scale study by our group showed that including different fecal phenotypes can increase the variability of results and should thus be considered (2). Therefore, the objective of this study was to investigate the bacterial communities in different fecal phenotypes shortly before and immediately after weaning in a cohort of 192 piglets. We compared the fecal microbiome composition of swab samples to that of feces of different colors and consistencies to evaluate whether fecal swab samples were similar to a certain fecal phenotype. We hypothesized that the bacterial composition associated with a specific phenotype would differ before and after weaning, but that indicator bacteria would be similar for a specific phenotype across ages.

## 2 Materials and methods

### 2.1 Animals, housing, and experimental procedures

The experiment was conducted at the pig facility of the research and teaching farm of the University of Veterinary Medicine of Vienna (Vetfarm, Vienna, Austria) using the progeny of 24 sows (*n* = 12 litters/replicate batch) in two replicated batches. The average litter size across replicate batches was 14.8 ± 1.65 [standard deviation (SD)] piglets. Sows and litters were housed in BeFree pens (BeFree, Schauer, Agrotonic, Prambachkirchen, Austria; 2.3 m × 2.6 m) throughout the suckling phase, similar to previously described conditions (5). Sows were kept loose during farrowing and lactation. Weaning occurred on the day of life (DoL) 28. Piglet’s health was monitored daily. Suckling piglets that received medical treatment were excluded from the study. During the suckling phase, piglets could suckle freely and had access to an additional milk replacer (Supplementary Table S1), which was offered in liquid form (after mixing the powder with lukewarm water; 1:5 w/v) in stainless steel piglet feed troughs from DoL3. The milk replacer was gradually replaced by the prestarter diet (Supplementary Table S1) from DoL21 to DoL23 and entirely replaced from DoL24. From DoL24 to DoL28 (preweaning), the prestarter was fed as a mash. Postweaning (starting on DoL28), the piglets received the prestarter diet at 100% in dry form until the end of the experiment.

For the present experiment, 192 clinically healthy (i.e., showing no clinical signs of illness) piglets (96 piglets/replicate batch) from 24 litters (4 piglets/litter) were selected on the weaning day. Except for one pair, two sibling pairs (one male and one female) were selected from each litter. The selected piglets were moved to the rearing barn and allotted to one out of four pens per replicate batch (3.3 m × 4.6 m; 12 piglets/pen) with equal distribution of sexes and litters and average body weight. Each pen was equipped with a heated box as a lying area, two hinged piglets’ drinking troughs at the aqua level, and a large plastic round feeder, which was filled manually up to three times per day according to the piglets’ appetite. Piglets were weighed at weaning (DoL28) and on DoL33 when collecting feces. Weighing and collection of fecal samples on DoL28 took place immediately after the separation of the sow and piglets. The piglets had free access to water and food throughout the experimental phase.

### 2.2 Monitoring of fecal consistency and collection of feces

Fecal consistency was monitored daily from DoL28 to DoL36 by visual inspection of both the piglets´ perianal region and the floor of the pen. Consistency was scored as follows: 5 (hard and dry balls), 4.5 (clearly defined shape with cracks, leaves no residue on the ground when picked up), 4 (clearly defined shape with cracks, leaves little residue on the ground when picked up), 3.5 (pasty; moist feces with distinct shape), 3 (pasty; moist feces with no cracks and no distinct shape), 2.5 (moist feces, with little consistency and no real shape), 2 (very wet but not liquid feces), 1.5 (liquid feces with minimal consistency), and 1 (entirely liquid feces). The scoring method was adapted from previous studies (2, 10) and adjusted for this study. Compared with previous studies (2, 10), the scoring system applied in the present study provided a more gradual classification (1–5 scale with intermediate values), allowing finer discrimination of subtle transitions in fecal consistency, which was tested and established before the start of the experiment. All visual inspections were performed by the same observer.

Fecal samples from weaned piglets were collected on DoL28 and 33 by rectal stimulation using the tip of a sterile cotton swab covered with veterinary gel to gently massage the internal sphincter and induce the defecation reflex. Defecated feces were collected into 15 mL tubes (Sarstedt AG & Co., Nümbrecht, Germany). Feces and swabs were placed together in the tube when only a small amount of fecal matter was obtained after rectal stimulation. The swab tip served as a backup for DNA extraction if the fecal amount was too small. In the absence of feces, only smeared cotton swabs were collected. The samples were kept on crushed ice during collection and then stored at −80 °C until further processing. The types of samples collected (feces or swabs), color (brown, yellow, or swab samples), and consistency (ball-shaped, liquid feces, or swab samples) of the feces were recorded. For the analysis of the fecal microbiota by phenotype “consistency,” fecal samples were categorized into two groups: (1) representing feces with a certain consistency and a score of 2 or higher (called “balls”); and (2) representing feces with almost no consistency and a score of 1–1.5 (called “liquid feces”). The number of samples collected for each category is listed in Supplementary Table S2.

### 2.3 DNA extraction, quantitative PCR, and 16S rRNA amplicon sequencing

Total DNA was extracted from piglet feces and swab samples using the DNeasy PowerSoil Pro Kit (Qiagen, Hilden, Germany), with modifications to the manufacturer’s protocol. Approximately 200 mg of feces or 30 mg of swab material was weighed for DNA extraction. The processing of the mixed samples depended on whether sufficient fecal material (200 mg) was present in the tube. If less than 200 mg of fecal material was collected, swabs were used for DNA extraction. After weighing, lysis buffer was added, and for the swab samples, an additional incubation step (1 h at 4 °C in 800 μL phosphate-buffered saline [PBS]) was performed to dissolve the fecal material and mucus from the cotton swabs. Subsequently, a heating step (10 min at 70 °C) was performed using a ThermoShaker (Analytik Jena GmbH, Jena, Germany) for all sample types. The samples were homogenized for 60 s at three intervals with intermittent cooling on ice using a FastPrep 24 5G instrument (MP Biomedicals, Santa Ana, CA, USA). After homogenization, the samples were processed according to the manufacturer’s protocol. To measure the DNA concentration of each sample, a Qubit DNA HS assay kit and Qubit 4 fluorometer (Thermo Fisher Scientific Inc., Waltham, MA, USA) were used.

Total bacterial gene copy numbers were quantified using previously described conditions and primers in the qTOWER real-time polymerase chain reaction (PCR) system (Analytik Jena GmbH, Jena, Germany) (2). Each reaction contained 10 μL, composed of 2.5 μL innuMIX qPCR DSGreen Standard (IST Innuscreen, Berlin, Germany), 300 nM (0.3 μL) each of forward and reverse primers, 6.4 μL diethyl pyrocarbonate (DEPC)-treated water (G-Biosciences, St. Louis, MO, USA), and 0.5 μL of DNA template (approximately 40 ng per reaction) in a 96-well plate in duplicate. The amplification program consisted of 2 min of initial denaturation at 95 °C, followed by 40 cycles of 95 °C for 30 s for denaturation and 60 °C for 60 s for primer annealing and elongation. Primer specificity was determined using melting curve analysis with increments of 0.1 °C/s between 60 and 95 °C. Standard curves were prepared from 10-fold serial dilutions (10^10^–10^3^ molecules/μL) of the purified and quantified PCR products using pooled DNA from fecal samples collected during the experiment. The final copy numbers were calculated using the following equation: (QM × C × DV)/(S × W), where QM is the quantitative mean copy number, C is the DNA concentration of each sample, DV is the dilution volume of the isolated DNA, S is the amount of DNA (ng), and W is the weight of the sample (g) subjected to DNA extraction. The amplification efficiencies (E = 10^(−1/slope)^) and coefficients of determination (linearity) are listed in Supplementary Table S3.

One aliquot of the DNA extract was sent to a commercial provider (Novogene Co. Ltd., Cambridge, UK) for 16S rRNA amplicon sequencing, including library preparation (NEBNext DNA Library Prep Kit for Illumina; New England Biolabs, Ipswich, MA, USA). Amplification of the V3-V4 region of the bacterial 16S rRNA gene was performed with the primers 341-F (5’-CCTAYGGGRBGCASCAG-3′) and 806-R (5’-GGACTACNNGGGTATCT AAT-3′) (11). The target sequencing depth was 30,000 reads per sample. Equimolar pools of samples were sequenced to generate 250 bp raw reads on the Novaseq 6,000 platform (Illumina) using a paired-end protocol. Demultiplexing and trimming of the raw sequences were performed using Novogene.

### 2.4 Bioinformatic analysis

The raw sequencing reads (FASTQ files) were processed using the Quantitative Insights Into Microbial Ecology, i.e., QIIME2 v2023.2 (12). Trimmed reads were imported, and read quality was initially inspected using FastQC (13). Sequence data were quality filtered using the q-score-joined plugin and 30 as the minimum acceptable Phred score, resulting in trimming lengths of 225 and 222 nucleotides for forward and reverse reads, respectively. Reads were merged and denoised into amplicon sequence variants (ASV) using the Divisive Amplicon Denoising Algorithm 2 (DADA2). Taxonomy was assigned to the ASV using a classify-sklearn naïve Bayes taxonomy classifier trained with the 341 F/806R primer set against the SILVA 138.2 99% reference database. A taxonomic table was built, and sequence reads were filtered, removing mitochondria and chloroplasts using the “filter-table” function. Alpha-diversity (Shannon index, Simpson index, and observed features) was analyzed using the R package phyloseq (version 1.48.0) after rarefaction to 29,279 reads, which represented the number of reads in the sample with the fewest reads. Boxplots for alpha-diversity metrics were obtained using the package ggplot2 (v 3.5.1). Sequence data were converted into relative abundance with the “qiime feature-table relative-frequency” function, using the final DADA2 table as input. The relative abundance output table was then filtered at the genus rank, multiplied by 100 to obtain percentages, and ranked from the highest to lowest average on Excel (v.2408). The 25 most abundant taxa were statistically analyzed (>1.1% of all reads).

### 2.5 Statistical analysis

Statistical analysis of growth performance data, daily monitoring of fecal consistency, and microbiome data were performed using SAS (version 9.4, SAS Inst. Inc., Cary, NC, USA). The residuals of the data for growth performance, fecal scores, total bacterial gene copies, alpha-diversity, and taxonomic bacterial composition were first analyzed for normality using the Shapiro–Wilk test in SAS. The data were transformed using the Box–Cox method and Transreg procedure in SAS if the residuals were not normally distributed, which was the case for the taxonomic bacterial composition. Subsequently, the normalized data were subjected to analysis of variance (ANOVA) using the MIXED procedure in SAS. Repeated measures were used to assess fecal scores and body weight data over time. The fixed effects consisted of replicate batch, sex, DoL, and their interaction. For body weight, litter size before weaning was used as a covariate. In the second model, repeated measures were used to assess the effects of fecal sample type, color, and consistency over time on total bacterial gene copies, relative abundance of genera, and alpha-diversity indices (Shannon and Simpson). The fixed effects consisted of replicate batch, sex, DoL, sample type (feces, swab), color (brown, yellow), consistency (balls, liquid feces), and two-way interaction with DoL. In both models, the piglets nested within the litter and pen represented the experimental unit. Degrees of freedom were approximated using the Kenward–Roger method. Differences among least squares means were computed using the pdiff statement. For total and relative bacterial abundance (genus level), Bonferroni correction was applied to adjust the raw *p* values for differences within the sample type, color, and consistency. Data were expressed as least squares means ± standard error of the mean (SEM). Differences were considered significant if *p* ≤ 0.05, and as a trend if 0.05 < *p* ≤ 0.10. In order to represent the change in relative abundance of the microbiome between the two sampling points (DoL28-33), the log_2_ fold changes were calculated from the relative abundance results of the >1.1% most abundant genera with significant phenotype × DoL interaction (*p* < 0.05) using the log2() function in R. Descriptive statistics for the feed intake of piglets were calculated using Excel (v.2408). Statistical assessment of Bray–Curtis dissimilarity matrices was performed using the ‘adonis2’ function, Permutational Multivariate Analysis of Variance (PERMANOVA), of the vegan package (v. 2.6–8) in R.

## 3 Results

### 3.1 Body weight and fecal consistency scores

**Figure 1:**
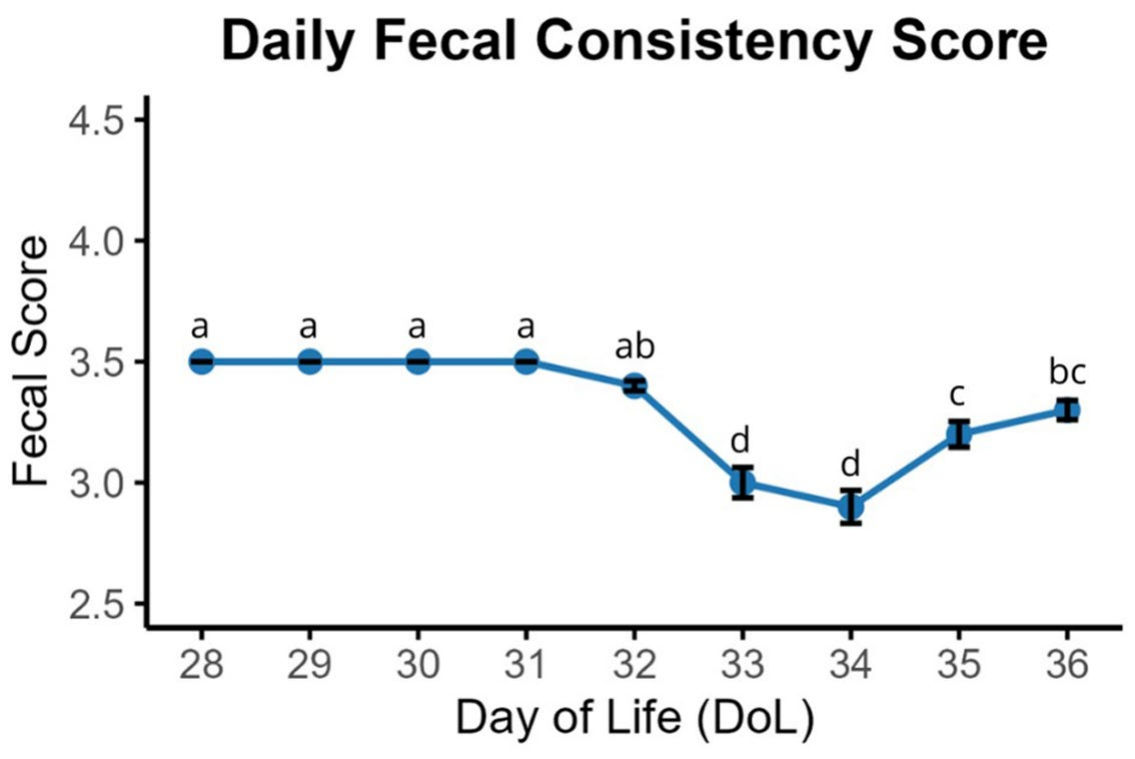
Daily monitoring of fecal consistency from DoL28 to DoL36. Consistency was scored as follows: 5 (hard and dry balls), 4.5 (clearly defined shape with cracks, leaves no residue on the ground when picked up), 4 (clearly defined shape with cracks, leaves little residue on the ground when picked up), 3.5 (pasty; moist feces with distinct shape), 3 (pasty; moist feces with no cracks and no distinct shape), 2.5 (moist feces, with little consistency and no real shape), 2 (very wet but not liquid feces), 1.5 (liquid feces with minimal consistency), and 1 (entirely liquid feces). (a–c) Means without a common superscript in the same row differ significantly (*p* < 0.05).

On the weaning day (DoL28), piglets had an average body weight of 7.6 ± 0.09 kg (SEM), which increased to 7.8 ± 0.09 kg (SEM) on DoL33 (*p* < 0.001). Fecal scores were assessed from DoL28 to DoL36 (Figure 1). The fecal score was 3.5 between DoL28 and DoL32 and decreased to 3.0 on DoL33 and DoL34, rising again to 3.0 and 3.5 on DoL35 and DoL36, respectively (*p* < 0.001). On DoL28, only two samples were classified as “liquid feces” in terms of consistency (Supplementary Table S2). Therefore, the results obtained for these samples should be interpreted with caution and should be exploratory. Piglets consumed, on average, 50 g (dry matter basis) of creep feed daily from DoL21 to DoL27 (preweaning) and 355 g (dry matter basis) of prestarter diet daily from DoL28 to DoL36 (postweaning) (Supplementary Table S4).

### 3.2 Differences in bacterial community between DoL28 and DoL33

**Figure 2:**
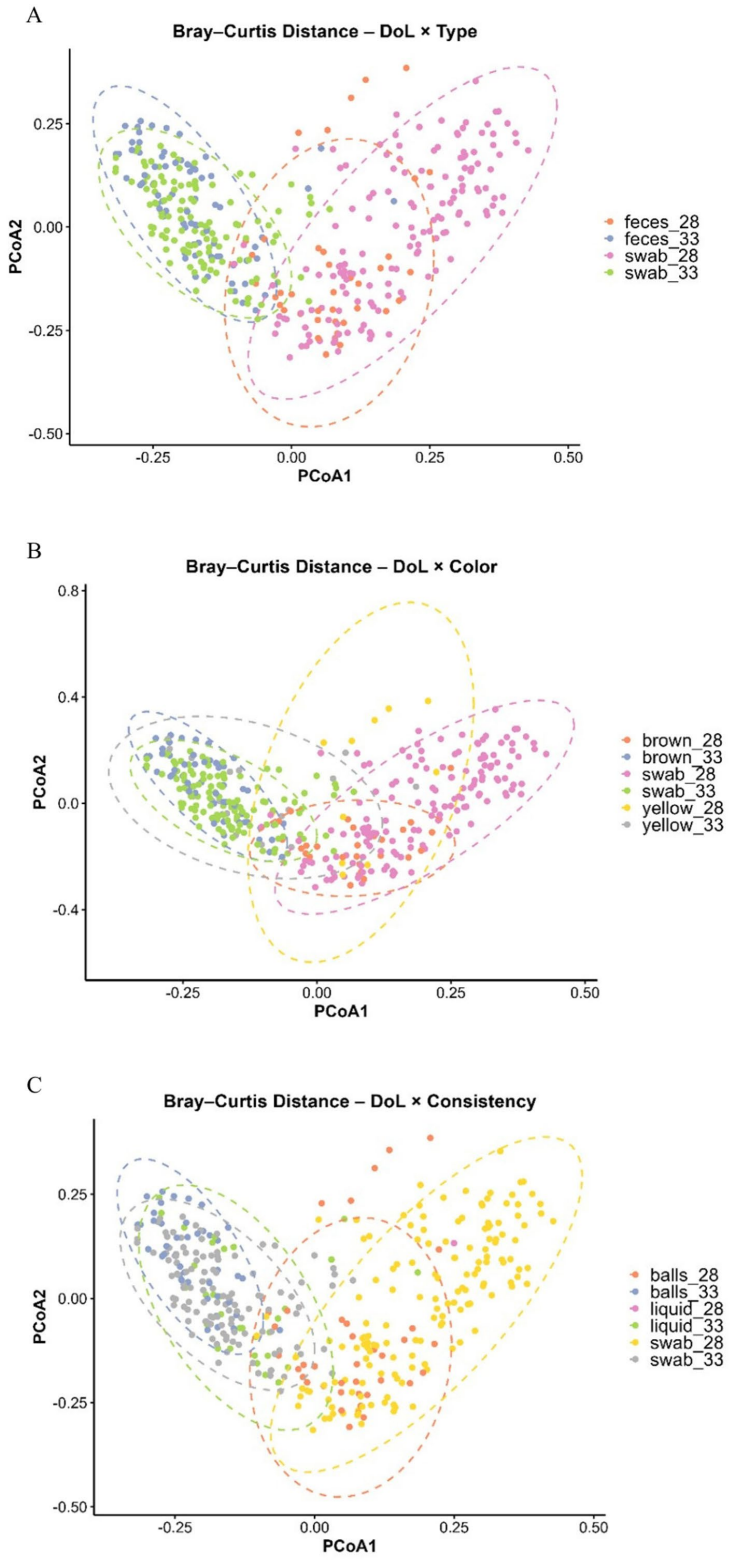
Principal coordinates analysis (PCoA) plot based on pairwise Bray–Curtis dissimilarities among bacterial communities in feces of weaned piglets across fecal sampling type **(A)**, color **(B)**, and consistency **(C)** at DoL28 and DoL33. Ellipses represent 95% confidence intervals.

**Figure 3:**
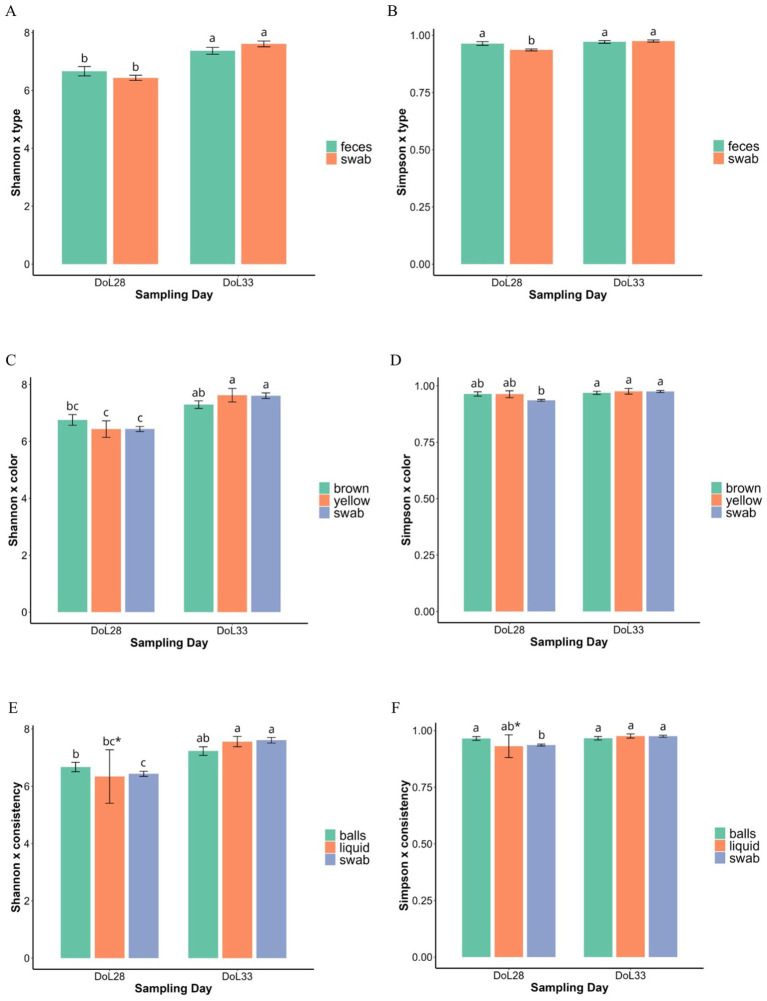
Shannon and Simpson indices in fecal samples of different types **(A,B)**, colors **(C,D)**, and consistencies **(E,F)** collected from weaned piglets at DoL28 and DoL33. Piglets were weaned at DoL28. *Only two piglets were marked with “liquid feces” at DoL28. (a–c) Means within the same row that do not share a common superscript differ significantly (*p* < 0.05).

**Figure 4:**
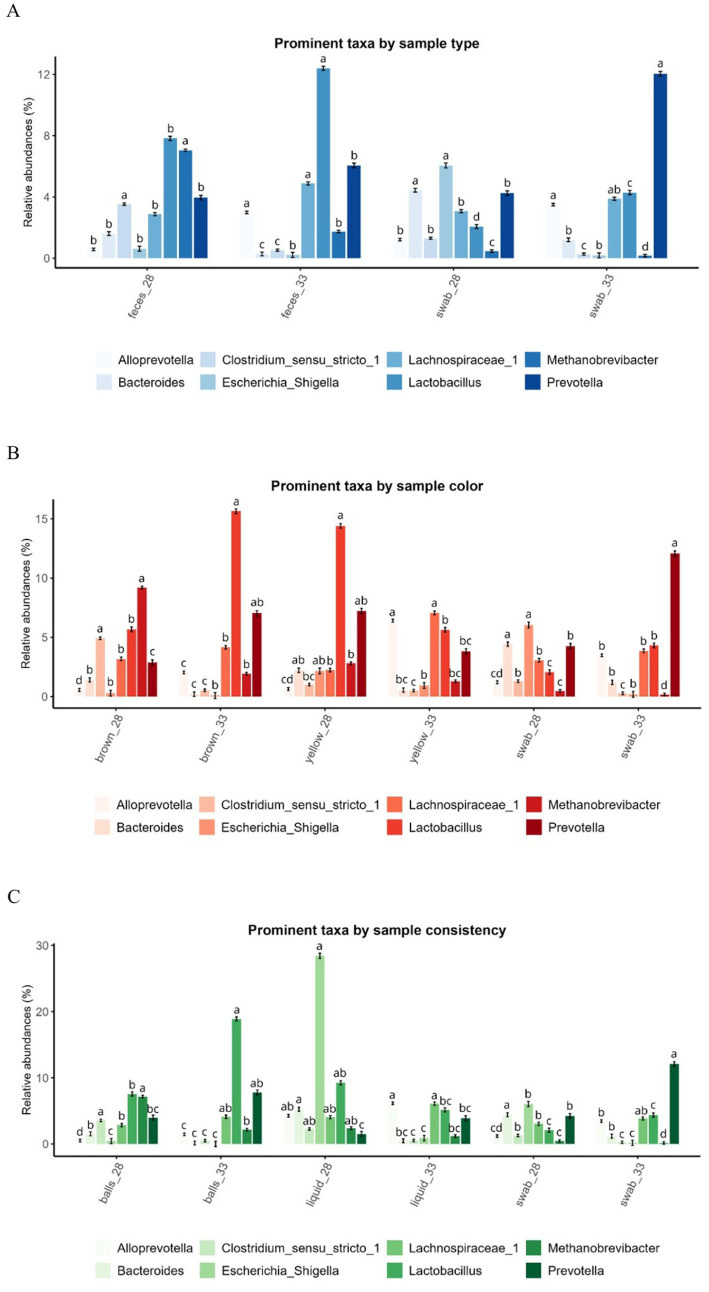
Relative abundances of the most prominent taxa by fecal sample type **(A)**, color **(B)**, and consistency **(C)** on DoL28 and DoL33. Data are presented as least squares means ± SEM. **(A–C)** Means within the same row, those that do not share a common superscript differ significantly (*p* < 0.05).

Across all fecal phenotypes, there was no difference in gene copy numbers between DoL28 and DoL33 (*p* > 0.05; Supplementary Table S5). Beta-diversity analysis (PERMANOVA) supported age-related bacterial communities in DoL28 and DoL33 (Supplementary Table S6), as shown in the Principal Coordinates Analysis (PCoA) plots in Figure 2. Alpha-diversity using the Shannon index increased from DoL28 to DoL33 (*p* < 0.05) (Figures 3A,C,E), whereas the Simpson index did not show major differences over time (*p* > 0.05) (Figures 3B,D,F). The day of life affected the relative abundances of many predominant bacterial genera (>1.1% relative abundance). On DoL28, *Escherichia, Methanobrevibacter, Fusobacterium, Helicobacter, and Clostridium* sensu stricto-1 were the predominant taxa, which were less abundant on DoL33 (*p* < 0.05). In contrast, the relative abundances of *Prevotella*, *Lactobacillus*, *Alloprevotella*, *Prevotellaceae* NK3B31, UCG-003 gut groups, and *Blautia* increased from DoL28 to 33 (*p* < 0.05) (Figure 4).

### 3.3 Differences in bacterial community between fecal sample types

**Figure 5:**
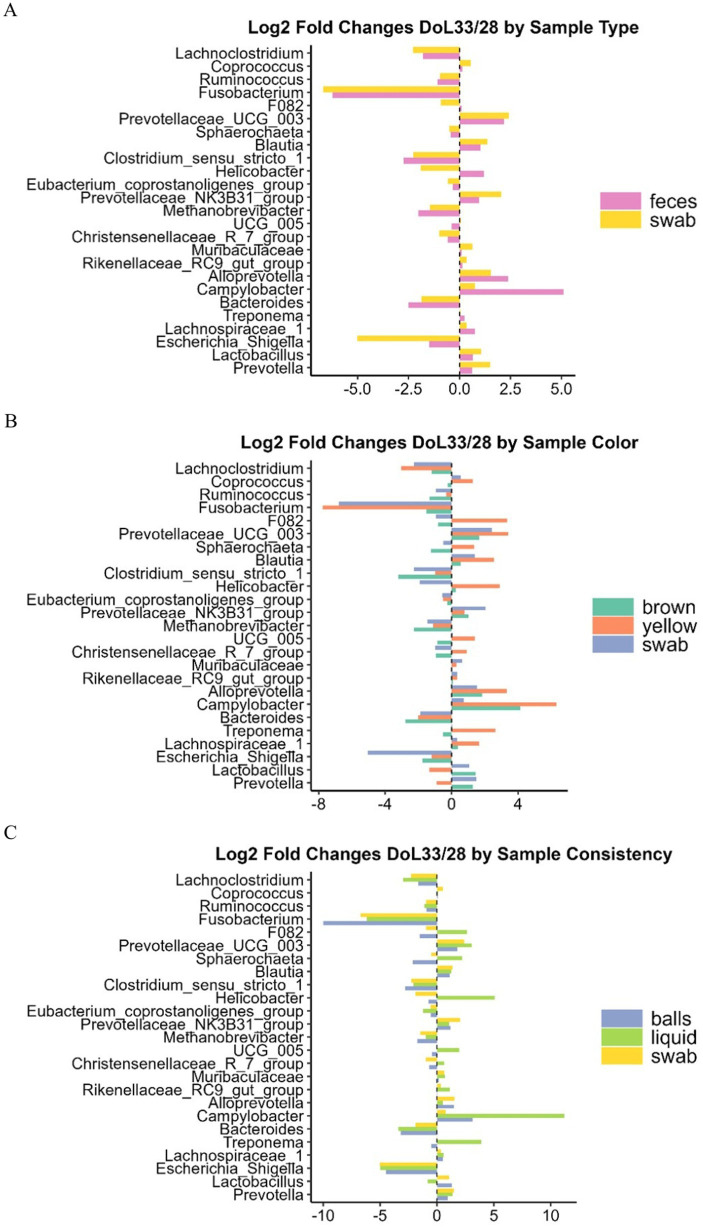
Log_2_ fold changes in relative abundance (% of all reads) of the most discriminant bacterial genera for type **(A)**, color **(B)**, and consistency **(C)** of fecal samples in weaned piglets at DoL28 and DoL33.

Feces contained 0.8- and 0.6-log units more gene copies per gram of sample than swab samples on DoL28 and DoL33, respectively (*p* < 0.001; Supplementary Table S5). In addition, the DoL× sample type interaction indicated a 0.3-log unit higher bacterial abundance in swab samples (but not in fecal samples) on DoL33 than on DoL28 (*p* = 0.038; Supplementary Table S5). The PERMANOVA (beta-diversity) indicated that bacterial communities differed on DoL28 and DoL33 in both fecal and swab samples (*p* < 0.001; Supplementary Table S6). However, the illustration in the PCoA plot only supported separate clustering of bacterial communities on DoL28 and DoL33, whereas there was a certain overlap in the bacterial communities between sample types at each of the two sampling time points (Figure 2A). Regarding alpha-diversity, the Shannon index was higher in both sample types (feces and swabs) on DoL33 than on DoL28 (*p* < 0.05), with no difference between the two sampling methods on the same day of life (*p* > 0.05; Figure 3A). On DoL28, the Simpson index was higher in fecal samples than in swab samples (*p* < 0.05; Figure 3B). The Simpson index increased in the swab samples from DoL28 to DoL33 to reach a similar diversity as that in fecal samples from DoL33 (*p* < 0.05; Figure 3B). The most discriminant taxa between sample types, along with their relative abundances, are shown in Figure 4A. The taxonomic composition showed sample type-associated differences in the relative abundances of 20 bacterial genera, which represented more than 75% of all reads (*p* < 0.05; Supplementary Table S7). Moreover, DoL × sample type interactions indicated that the effect of the sample on the relative abundance of eight genera (>20% of all reads) differed between the two time points. On DoL28, fecal samples had a higher relative abundance of *Methanobrevibacter* and *Clostridium* sensu stricto-1 than swab samples (*p* < 0.001). In contrast, swab samples had higher relative abundances of *Escherichia*, *Helicobacter,* and *Fusobacterium* than fecal samples on DoL28 (*p* < 0.05). On DoL33, fecal samples were characterized again by a higher relative abundance of *Methanobrevibacter* compared to swab samples (*p* < 0.001). Swab samples, in turn, comprised more *Prevotella*, *Prevotellaceae* NK3B31*, Erysipelotrichaceae* UCG-003 gut group, and *Helicobacter* on DoL33 than fecal samples (*p* < 0.05). Log_2_ fold changes illustrated the alterations in abundances of bacterial genera between the two sample types from DoL28 to DoL33 (Figure 5A). Results showed that the relative abundances of *Prevotella* and *Lactobacillus* increased by 1.5- and 1.0-fold from DoL28 to DoL33 in swab samples and by 0.6- and 0.7-fold in fecal samples, respectively. *Campylobacter*, *Alloprevotella*, and *Lachnospiraceae*-1, in turn, increased by 5.1-, 2.5-, and 0.8-fold, respectively, from DoL28 to DoL33 in feces, but only by 0.7-, 1.5-, and 0.3-fold in swabs. The relative abundances of *Escherichia*, in turn, decreased by 5.0-fold in swab samples and 1.5-fold in feces samples from DoL28 to DoL33 (*p* < 0.05). Moreover, the relative abundances of *Methanobrevibacter* and *Bacteroides* decreased by 2.0- and 2.5-fold, respectively, from DoL28 to DoL33 in fecal samples and by 1.5- and 1.9-fold, respectively, in swab samples (*p* < 0.05).

### 3.4 Differences in bacterial community between fecal color

The DoL × fecal color interaction indicated that on DoL28, brown and yellow feces contained similar gene copies, which were 0.9- and 0.7-log units higher than those in the swab samples (*p* < 0.05; Supplementary Table S5). In contrast, in DoL33, brown feces contained 0.6- and 0.7-log units more gene copies than yellow feces and swab samples (*p* < 0.05). According to the PERMANOVA (Supplementary Table S6), feces of different colors comprised diverging bacterial communities (*p* < 0.001). The PCoA plot showed different clustering among bacterial communities according to the sampling time points, with less clustering for fecal color and swab samples (Figure 2B). While yellow and swab samples comprised a higher diversity (Shannon and Simpson) on DoL33 than on DoL28, brown feces contained similar bacterial diversity on both sampling days (Figures 3C,D). The most different genera for fecal color and swabs by relative abundance are shown in Figure 4B. Feces of different colors (brown and yellow) and swab samples differed in the relative abundance of 20 bacterial genera (>75% of all reads; *p <* 0.05; Supplementary Table S8). Moreover, DoL × sample color interactions indicated that 16 bacterial genera (>40% of all reads) did not show the same abundance in yellow and brown feces or swabs on DoL28 and DoL33 (*p <* 0.05). On DoL28, brown fecal samples had a higher relative abundance of *Methanobrevibacter*, *Oscillospiraceae* UCG-005*, Clostridium* sensu stricto-1, *Blautia, Ruminococcus*, and *Coprococcus* than yellow fecal samples and swabs (*p* < 0.05). In contrast, yellow fecal samples had higher relative abundances of *Lactobacillus* and *Fusobacterium* compared to brown feces on DoL28 (*p* < 0.05). On DoL33, brown feces had a higher relative abundance of *Lactobacillus* than yellow feces (*p* < 0.05). Yellow samples comprised more *Lachnospiraceae*-1 and *Alloprevotella* than brown feces on DoL33 (*p* < 0.05). The relative abundance of *Prevotella* was similar in swab samples and brown feces but lower in yellow feces on DoL33. Likewise, *Campylobacter* was similarly abundant in swab and yellow feces and was higher than in brown feces on DoL33 (*p* < 0.05; Supplementary Table S8). These findings were further supported by the log_2_ fold change analysis comparing bacterial abundance between DoL28 and DoL33 using sample color as the variable (Figure 5B). The results highlighted that the abundance of *Prevotella* and *Lactobacillus* increased 1.3- and 1.5-fold from DoL28 to DoL33 in brown fecal samples, respectively, and by 1.5- and 1.0-fold in swab samples, respectively, but decreased by 0.9- and 1.4-fold in yellow feces (*p* < 0.05). *Methanobrevibacter* tended to decrease by 2.3-fold in brown fecal samples and by 1.1- and 1.5-fold in yellow fecal and swab samples, respectively (*p* < 0.05). The relative abundances of *Escherichia, Fusobacterium*, and *Bacteroides* decreased by 1.8-, 1.5-, and 2.8-fold, respectively, in brown fecal samples; by 1.2-, 7.8-, and 2.0-fold in yellow fecal samples; and by 5.0-, 6.8-, and 1.9-fold in swabs from DoL28 to DoL33, respectively (*p* < 0.05).

### 3.5 Differences in bacterial community between fecal consistencies

Regarding the total bacterial gene copy numbers, ball-shaped and liquid feces had similar gene copy numbers, which were 0.8 log units higher than those of the swab samples on DoL28 (*p* < 0.001; Supplementary Table S5). On DoL33, in turn, ball-shaped feces comprised 0.6 and 0.8 log units more than liquid and swab samples (*p* < 0.05). The PERMANOVA supported different clustering of bacterial communities according to sample consistency (Supplementary Table S6), whereas the illustration in the PCoA plot showed that bacterial communities overlapped to a certain degree among consistency types and swab samples on both DoLs (Figure 2C). Ball-shaped feces contained a more diverse bacterial community than swab samples (*p* < 0.05; Figures 3E, F). Liquid feces exhibited similar bacterial diversity (Shannon and Simpson indices) to ball-shaped feces and swabs on DoL33. The most discriminative genera for fecal consistency by relative abundances are presented in Figure 4C. Feces of different consistencies (ball-shaped, liquid, and swab) showed differences in the relative abundances of 18 bacterial genera (>70% of all reads) (*p <* 0.05; Supplementary Table S9). Moreover, five bacterial genera (>24% of 1.7% analyzed reads) had different abundances at the two sampling time points in feces of different consistencies, as indicated by the DoL × sample consistency interactions (*p <* 0.05). On DoL28, ball-shaped feces had a higher relative abundance of *Oscillospiraceae* UCG-005 than swab samples (*p* < 0.05), but not on DoL33. The two liquid fecal samples collected on DoL28 had substantially higher relative abundances of *Escherichia* than the firmer feces and swab samples (*p* < 0.05). However, both liquid feces and swab samples contained more *Bacteroides* than ball-shaped feces on DoL28, whereas ball-shaped feces comprised more *Methanobrevibacter* than swab samples (*p* < 0.05). On DoL33, ball-shaped feces had a higher relative abundance of *Lactobacillus* than liquid samples and swabs (*p* < 0.05). Ball-shaped and swab samples contained more *Prevotella* than liquid feces on the same day (*p* < 0.05). In contrast to DoL28, the relative abundance of *Escherichia* was similar on DoL33 among the ball-shaped, liquid, and swab samples (*p* < 0.05; Supplementary Table S9). Comparison of the relative abundances of bacterial genera based on log_2_ fold changes between DoL28 and DoL33 (Figure 5C) showed a general decrease in *Escherichia* in all phenotypes between 4.5- and 5-fold log_2_ changes. Moreover, the presentation of the log_2_ fold changes among ball-shaped, liquid, and swab samples demonstrated that in liquid feces, the abundance of *Treponema*, *Campylobacter*, and *Helicobacter* increased by 3.9-, 11.2-, and 5.0-fold, respectively, from DoL28 to DoL33 (*p* < 0.05), whereas for ball-shaped feces and swab samples, changes in these abundances were less drastic from DoL28 to DoL33. In contrast, the abundance of *Fusobacterium* dropped by 10.0-fold from DoL28 to DoL33 in ball-shaped feces but only 6.2-fold in yellow feces (*p* < 0.05).

## 4 Discussion

A better understanding of gut microbiota colonization patterns in piglets helps design monitoring and intervention strategies to support gut health during weaning. During weaning, stress and immature gut functions often alter fecal appearance, even in the absence of pathogens. Different types of samples are used for fecal microbiota research, including feces, biopsy tissue, mucosal samples from the cavity wall, and intestinal lavage fluid (14). These samples are obtained via direct fecal collection, rectal swabs, endoscopic biopsies, cytological brushing, mucosal swabs, and aspiration of intestinal luminal lavage. There is still a debate over which type of sample constitutes a representative alternative to fecal samples (e.g., mucosal swabs), particularly in cases where obtaining feces is difficult (2, 4, 8, 9, 14). Moreover, sampling strategies targeting mucosal surfaces, such as cytological brushes, may be more representative of epithelial-associated microbiota than mucosal swabs, but results can vary depending on the sampling depth (14). However, cytological brushes are more invasive and may be less practical for longitudinal or large-scale studies. Therefore, in the present study, we compared the microbiota composition in feces and rectal swabs as a widely used and validated sampling method in piglets, representing a minimally invasive approach for microbiota assessment that is feasible at the farm level (4, 8).

A previous small-scale study from our group indicated that fecal bacterial microbiota diverged in feces of similar consistency but different colors in suckling and newly weaned piglets (2). Therefore, in the present study, we screened the bacterial microbiota in the feces of a large cohort of piglets before and 5 days after weaning, the period when piglets are highly susceptible to developing postweaning diarrhea. The present results confirm that bacterial communities vary according to the type of sample collected and the phenotypic features of feces, with variation before and after weaning. In this respect, the data demonstrate that genera containing disease agents were not substantially increased in liquid feces on DoL33. Instead, a lower abundance of *Lactobacillus* was the characteristic difference in liquid feces compared to ball-shaped feces on DoL33, which should be distinguished from pathogen overgrowth. The current data further suggest that differentiating feces according to their phenotype may also provide information about plant-based food intake and intestinal function around weaning, which should be followed up on in the future (2). In the present study, fecal samples were divided into broad categories of fecal phenotypes, without considering intermediate appearances. Therefore, when interpreting the results, it should be considered that there is more natural variability, which may be better captured using digital tools in the future. Moreover, targeting the V3–V4 region of the 16S rRNA gene allows taxonomic resolution primarily at the genus level, limiting the discrimination between species or strains with different functional or pathogenic roles (15).

Furthermore, a comparison between swab samples and alternative sampling tools, such as cytological brushes, is warranted to better contextualize the chosen approach. As highlighted in a recent study, sampling strategies targeting mucosal surfaces, including the use of cytological brushes, may enable a more effective recovery of epithelial-associated microbiota, although the results can vary depending on the sampling depth and anatomical site (14). However, these approaches are generally more invasive and less practical, particularly in longitudinal or large-scale studies. In contrast, rectal swabs are widely used and validated in piglets, representing a more feasible and minimally invasive approach for microbiota assessment (4, 8). Therefore, while cytological brushes may offer advantages for targeted mucosal investigations, their added value over swabs in rectal sampling of piglets has not yet been clearly established.

The alterations in fecal consistency observed during daily monitoring were consistent with those reported in the literature during the first week after weaning (1, 7). The fact that feces were generally firm until DoL32 may be due to a reduced passage rate caused by low feed intake in the first days postweaning. Although piglets were offered the prestarter diet as creep feed before weaning in the present study, we can assume that weaning caused a drop in feed intake due to stressors such as relocation, separation from the sow, regrouping, and relying entirely on solid feed for nutrition (16, 17). From DoL33 to DoL36, the increased occurrence of softer feces probably reflected the resumption of feed intake and change in feed, specifically the lack of sow’s milk. With mature digestive functions and higher feed intake, more substrate reaches the large intestine from DoL33, where it is readily fermented (16, 17). The microbial metabolites produced may have osmotic effects (2). Moreover, the observed alterations in the bacterial community from DoL28 to DoL33 probably caused an immunological reaction in the gut mucosa that resulted in increased secretory action in the gut (16, 17). Consequently, the piglets defecated softer feces.

Piglets were weaned on DoL28. Therefore, the analyzed fecal bacterial communities on this day were still representative of a milk-based diet (2, 5). Accordingly, the relative abundances of *Bacteroides, Clostridium* sensu stricto-1*, Ruminococcus*, and *Fusobacterium*, genera with glycoside hydrolase enzyme activities that utilize milk glycans (18), were higher on DoL28 but declined on DoL33 owing to a lack of substrate. By DoL33, in turn, the community was already adapted to the plant-based prestarter diet and dominated by bacteria specialized in fermenting complex plant carbohydrates (2, 5, 17). Accordingly, taxa, such as *Alloprevotella*, *Lactobacillus*, *Oscillospiraceae*, and *Lachnospiraceae*, increased in their abundance on DoL33 compared to DoL28. Gene copy numbers were almost similar on DoL28 and DoL33, suggesting that the changes in the bacterial community mainly occurred via alterations in the taxonomic composition, but not in the overall bacterial load. Nevertheless, consumption of only the cereal-based prestarter diet promoted the diversification of the fecal bacterial community in the 5 days after weaning, as indicated by higher alpha-diversity indices (Shannon and Simpson), thus aligning with previous findings for newly weaned pigs (19–21). Across sample types and phenotypes, the PCoA plots illustrated that age was the dominant driver for the separation of bacterial communities, whereas PERMANOVA indicated significant effects of sampling day and sample type or phenotype. A possible explanation relies on the fact that PERMANOVA can detect subtle differences in high-dimensional space, whereas PCoA mainly emphasizes major sources of variation (21–23).

We first analyzed the data for sample type, irrespective of color and consistency. In line with previous observations (19–21), the present data support the differences between fecal and swab samples, highlighting the influence of the sampling method on results regarding the fecal microbiota composition. Accordingly, swab samples may more closely reflect the mucosal community and less accurately reflect the bacterial community in feces. In contrast, Choudhury et al. (4) concluded from their results for suckling piglets between DoL3 and DoL20 that the bacterial communities associated with swabs and feces were comparable. One possible explanation for the differing results could be the varying amounts of digesta residue in the rectal mucosa sampled with the swabs. The fact that swab and fecal samples were equally diverse in the present study may suggest similar favorable growth conditions and microbial interactions at the two intestinal niches. Gene copy numbers, in turn, suggested less bacterial biomass in the rectal mucosa compared to feces. This may be related to the availability of nutrients in the large intestinal contents and/or to specific characteristics of the bacteria that allow them to adhere to and utilize mucus as a substrate (2, 24). The observation that differences in gene copies in fecal and swab samples were greater preweaning than postweaning may indicate a potentially thicker mucus layer at the rectal mucosa due to the greater amount of residual fiber in digesta, serving as a bacterial substrate and fostering bacterial growth. The taxonomic composition of feces and the swabs reflected the two intestinal niches and was similar to previous studies (4, 9, 25). Accordingly, species with mucin-degrading capabilities within *Escherichia*, *Bacteroides*, *Campylobacter*, *Helicobacter*, and *Fusobacterium* (2, 26, 27) may explain the higher abundance of these genera in swab samples than in feces. Although some of these taxa comprise pathobionts, they belong to the commensal bacterial community in the porcine gut mucosa (2, 26). We anticipated that the residual plant polysaccharides from the prestarter diet would promote the relative abundance of *Prevotella* in feces after weaning, similar to previous studies (2, 5, 28). However, *Prevotella* was less abundant in feces than in swab samples on DoL33 compared to DoL28, indicating that the mucin composition at the rectal mucosa fostered mucin-degrading species within *Prevotella* (29). Moreover, other taxa with similar substrate preferences may have outcompeted *Prevotella*. Accordingly, highly abundant *Lactobacillus* and other lower-abundant genera within the families *Lachnospiraceae* and *Prevotellaceae* seem to be the major carbohydrate-fermenting taxa in feces (5, 28). Differences in cross-feeding relationships due to diet-induced changes in metabolite availability may explain the changes in the relative abundances of other genera from DoL28 to DoL33.

Next, the bacterial communities in piglet feces were compared based on their phenotype and additionally compared to swab samples to determine whether the bacterial community associated with a specific phenotype more closely resembled the community in the swab samples. During the suckling phase, the yellow color of piglets’ feces was an indicator of their milk intake (7, 30, 31). When piglets began eating the plant-based prestarter diet, the fecal color turned brown, a common observation that can be linked to changes in bile secretion and microbial action on bile and plant feed residues (30, 31). However, the intake of the prestarter diet was low across all litters before weaning, and some piglets may not have consumed creep feed. Therefore, changes in fecal color may be a useful indicator for monitoring the solid feed intake of piglets. Accordingly, brown feces are distinguished from yellow feces by higher abundances of *Ruminococcus, Coprococcus*, and *Christensenellaceae* R-7 group, which comprise members involved in the degradation of plant-derived carbohydrates (32, 33), and thus may be indicative of plant glycan consumption on DoL28. If cross-feeding relationships in the porcine large intestine resemble those described in the bovine rumen, hydrogen produced during carbohydrate fermentation by *Ruminococcus* and *Christensenellaceae* R-7 groups may be utilized by hydrogenotrophic methanogens such as *Methanobrevibacter* (34), explaining the high abundance of the latter genus on DoL28. Interestingly, *Lactobacillus* was the predominant genus in yellow feces on DoL28, probably associated with milk-glycan fermentation (2, 5). Postweaning, brown feces were characterized by a similarly high abundance of *Lactobacillus* as yellow feces before weaning. Due to the change in dietary glycan composition from pre- to postweaning, it can be assumed that different species were present on DoL28 and DoL33. Piglets were fed the same preweaning diet postweaning, which should have had the same impact on fecal color. It is possible that due to the removal of fatty sow milk and very low intake of the prestarter diet, bile production in piglets with yellow feces declined more strongly during the days immediately after weaning than in piglets with brown feces. Microbial degradation of bilirubin, which is a component of bile, gives feces its distinctive color (35). Very low feed intake may have starved *Lactobacillus* in piglets with yellow feces, and after resumption of feed intake, *Lachnospiraceae*-1 and *Alloprevotella* may have partially replaced the niche of *Lactobacillus* in yellow feces in DoL33. Only the higher abundance of the pathobiont *Campylobacter* in yellow feces on DoL33 might be indicative of disturbances in gut homeostasis (36, 37), which could also be associated with more mucus in feces.

Regarding consistency, feces were categorized into two groups: liquid and ball-shaped feces, as these two groups are easily distinguishable in the barn. It is important to note that on DoL28, only a very few piglets had liquid feces, which seemed to be associated with *Escherichia*. We anticipated a lower gene copy number in liquid feces due to the higher water content and lower diversity as a sign of a less stable bacterial community. The latter was not confirmed by the present results for DoL33. Consistent with the etiology of postweaning diarrhea (29, 38), we also expected a higher abundance of *Escherichia* in liquid feces on DoL33 than in ball-shaped feces. However, this was not the case in this study. Instead, liquid feces were characterized by alterations in the abundance of carbohydrate-fermenting taxa (i.e., less *Lactobacillus* but more *Alloprevotella* and *Prevotellaceae* UCG-003) and an increase in *Campylobacter* and *Treponema,* which have been previously associated with diarrhea in pigs (36, 37). Accordingly, *Treponema* was a discriminant genus for liquid feces on DoL34 of life in our previous study (2). This increase may be related to the reduced abundance of *Lactobacillus*, whose regulatory activity normally suppresses pathogenic bacteria through bacteriocin production (39). However, none of the piglets with liquid feces required antibiotic treatment. In order to clarify the cause of liquid feces (i.e., pathogens or resumption of feed intake), it may be helpful to determine the expression of virulence factors in feces, as the bacterial composition did not provide a clear answer. When comparing the bacterial load and community in the swab samples with those of the two types of consistencies and colors, there were few similarities, indicating that swab samples taken from piglets do not necessarily resemble the bacterial community in the feces of a specific phenotype.

Although age was the dominant driver of microbiota maturation, phenotypic differences in feces were clearly associated with varying taxonomic compositions, suggesting their utility as accessible markers of gut health. As a next step, further studies should focus on assessing the association of fecal phenotypes with virulence factors and fecal markers for gut integrity. In practice, monitoring fecal phenotypes could help identify piglets at risk, enabling earlier interventions to improve animal welfare and reduce antimicrobial use. In gut microbiome studies, consideration of fecal phenotype may help reduce inter-animal variations in treatment groups.

## 5 Conclusion

This study highlights how sampling day, sample type, and fecal phenotypes are associated with different gut microbiota in weaned piglets. The microbial profiles shifted between DoL28 and DoL33, indicating rapid community transitions during the early postweaning phase. Clear differences emerged between fecal and rectal swab samples, with swabs potentially aligning more closely with mucosa-associated microbiota, while fecal samples reflected luminal communities. Importantly, fecal color and consistency were linked to distinct bacterial signatures, suggesting that visual phenotyping may capture meaningful biological variation. Our findings underscore the importance of selecting appropriate sample types and integrating fecal phenotypes when interpreting microbiome data. Such an approach may enhance the ability to detect early dysbiosis, facilitate the evaluation of gut health, and improve monitoring strategies for piglets during the critical weaning period.

## Data Availability

The datasets presented in this study can be found in online repositories. The names of the repository/repositories and accession number(s) can be found at https://www.ncbi.nlm.nih.gov/, PRJNA1424701.
