# Supplementary material for: Characterization of fecal bacterial microbiomes according to fecal color, consistency, and sample type in piglets before and after weaning
Source: Front Vet Sci. 2026 May 22;13:1815748. doi: 10.3389/fvets.2026.1815748 (PMC13236532; doi:10.3389/fvets.2026.1815748)
Supplement: Supplementary file 1 [file Table_1.DOCX]

Characterization of bacterial microbiomes according to fecal color, consistency, and type of sample in piglets before and after weaning

Supplementary Material

**Supplementary Table S1.** Analyzed nutrient composition of feed for suckling and weaned piglets.

| **Item** | **Milk replacer^1^** | **Prestarter diet^2^** |
| --- | --- | --- |
|  | | |
| Dry matter (DM) | 918 | 907 |
| Crude protein (g/kg DM) | 210 | 191 |
| Crude ash (g/kg DM) | 70 | 45 |
| Neutral-detergent fiber (g/kg DM) | 64 | 140 |
| Acid-detergent fiber (g/kg DM) | 22 | 61 |
| Acid-detergent lignin (g/kg DM) | 7 | 11 |
| Ether extract (g/kg DM) | 103 | 84 |
| Nitrogen-free extract (g/kg DM) | 598 | 627 |
| Starch (g/kg DM) | 211 | 370 |
| Sugar (g/kg DM) | 265 | 115 |
| Metabolizable Energy, MJ/kg DM^3^ | 16.63 | 15.53 |

^1^Weanplus-4, Startix, Voorthuizen, The Netherlands. Ingredient composition: whey powder, starch, soy protein, plant protein, plant oil (coconut oil and palm oil). Vitamin and mineral composition per kg feed: 25,000 IU of vitamin A, 8,000 IU of vitamin D3, 200 mg of vitamin E, 140 mg of Cu as chelate of glycine hydrate, 108 mg of Fe as chelate of glycine hydrate, 3 mg of I as calcium iodate, 115 mg of Zn as chelate of glycine hydrate, 35 mg of Mn as glycine chelate, 0.3 mg of Se as sodium selenite. Technological additives: 5 g of citric acid, 1 g of calcium formiate. Mixing ratio: 200 g of powder mixed into 1 L of 40 °C warm water.

^2^Ferkelabsetzkorn OGT, Garant-Tiernahrung GmbH, Pöchlarn, Austria. Ingredient composition: oat flakes, barley, wheat, whey powder, soy protein concentrate, wheat bran, sucrose, soybean meal, soy oil, corn gluten, L-cellulose, monocalcium phosphate, fish oil, sodium chloride, magnesium phosphate, calcium carbonate, and molasses. Vitamin and mineral composition per kg feed: 16,000 IU of vitamin A, 2,000 IU of vitamin D3, 200 mg of vitamin E, 120 mg Fe as iron (II) sulfate, 140 mg Cu as copper (II) sulfate, 120 mg Zn as zinc sulfate, 60 mg Mn as manganese (II) oxide, 1.5 mg I as calcium iodate, 0.5 mg Se as sodium selenite. Technological additives: 1,000 IU of phytase, 1,500 EPU of xylanase, 11 mg of beta hydroxy acid, 21 mg of butylated hydroxytoluene, 11 mg of propyl gallate.

^3^Calculated according to the DLG (Deutsche Landwirtschafts-Gesellschaft) guidelines for pig feed [1].

**Supplementary Table S2.** Number of samples collected for each category on DoL28 and DoL33.

| n. of samples | | **DoL28** | **DoL33** |
| --- | --- | --- | --- |
| *type* | feces | 39 | 74 |
|  | swabs | 153 | 118 |
| *color^1^* | brown | 28 | 53 |
|  | yellow | 11 | 21 |
| *consistency* | balls | 38 | 43 |
|  | liquid | 2 | 30 |

^1^Color and consistency were assessed exclusively on fecal samples, while swab samples were marked as “swabs” for both color and consistency.

**Supplementary Table S3.** Oligonucleotide primers for total bacteria used for quantitative PCR.

| **Group** | **Primer sequence (5’ to 3’)**^1^ | **R^2^** | **Efficiency (%)** | **Amplicon size (bp)** | **Reference** |
| --- | --- | --- | --- | --- | --- |
|  | | | | | |
| Total bacteria | F: CCTACGGGAGGCAGCAG  R: ATTACCGCGGCTGCTGG | 0.999 | 96.9 | 193 | [2] |

^1^F, forward primer; R, reverse primer.

**Supplementary Table S4.**Descriptive statistics for average daily creep feed and prestarter feed intake (consumed amount) during the suckling and post-weaning period.*

| Daily intake (kg dry matter) | Mean | SE | Minimum | Maximum | Median |
| --- | --- | --- | --- | --- | --- |
| DoL 21-27 | 0.049 | 0.002 | 0.029 | 0.059 | 0.055 |
| DoL 28-36 | 0.355 | 0.024 | 0.269 | 0.521 | 0.323 |

*Creep feed consumption was estimated at litter level (average 14.8 piglets/litter). DoL, day of life; SE, standard error of the mean. Piglets transitioned from the milk replacer to the prestarter from DoL21 to DoL23 and were offered the prestarter diet to 100% from DoL24.

**Supplementary Table S5.** Differences in gene copy numbers (log_10_ gene copies/g sample in feces of weaning piglets on day 28 and day 33 of life**.**

| **Variable** | **Day of life (DoL)** | |  | ***p-value*** | | |
| --- | --- | --- | --- | --- | --- | --- |
| **Type** | **28** | **33** | **SEM** | **DoL** | **Type** | **DoL×Type** |
| Feces | 10.4a | 10.5a | 0.052 | 0.001 | <0.001 | 0.038 |
| Swab | 9.6c | 9.9b |  |  |  |  |
| **Color** | **28** | **33** | **SEM** | **DoL** | **Color** | **DoL×Color** |
| Brown | 10.5a | 10.6a | 0.078 | 0.577 | <0.001 | 0.004 |
| Yellow | 10.3a | 10.0b |  |  |  |  |
| Swab | 9.6c | 9.9b |  |  |  |  |
| **Consistency** | **28** | **33** | **SEM** | **DoL** | **Consistency** | **DoL×Consistency** |
| Balls | 10.4b | 10.7a | 0.124 | 0.509 | <0.001 | 0.485 |
| Liquid | 10.4b* | 10.1b |  |  |  |  |
| Swab | 9.6d | 9.9c |  |  |  |  |

Values are presented as least squares means ± standard error of the mean (SEM). At each time point (DoL), 192 piglets (96 piglets/replicate batch) were sampled. Weaning occurred on DoL28.

a,b,c,d means without a common superscript in the same row differ (p<0.05).

*Only 2 piglets were marked with “liquid feces” on day 28 of life.

**Supplementary Table S6.** Permutational multivariate analysis of variance for bacterial communities in fecal samples collected on day 28 and 33 of life.

| **Day of Life** | **Variables** | **df** | **Sum of squares** | **R^2^** | **F** | ***p-*value** |
| --- | --- | --- | --- | --- | --- | --- |
| *28* | *type* | 1 | 3.413 | 0.052 | 10.365 | 0.001 |
|  | Residual | 190 | 62.562 | 0.948 |  |  |
|  | Total | 191 | 65.974 | 1.000 |  |  |
| *28* | *color* | 2 | 4.453 | 0.067 | 6.839 | 0.001 |
|  | Residual | 189 | 61.522 | 0.932 |  |  |
|  | Total | 191 | 65.974 | 1.000 |  |  |
| *28* | *consistency* | 2 | 3.723 | 0.056 | 5.651 | 0.001 |
|  | Residual | 189 | 62.251 | 0.943 |  |  |
|  | Total | 191 | 65.974 | 1.000 |  |  |
| *33* | *type* | 1 | 2.565 | 0.042 | 8.337 | 0.001 |
|  | Residual | 190 | 58.453 | 0.958 |  |  |
|  | Total | 191 | 61.018 | 1.000 |  |  |
| *33* | *color* | 2 | 3.342 | 0.054 | 5.475 | 0.001 |
|  | Residual | 189 | 57.676 | 0.945 |  |  |
|  | Total | 191 | 61.018 | 1.000 |  |  |
| *33* | *consistency* | 2 | 4.242 | 0.069 | 7.061 | 0.001 |
|  | Residual | 189 | 56.776 | 0.930 |  |  |
|  | Total | 191 | 61.018 | 1.000 |  |  |

df: degrees of freedom; DoL: day of life; F: F-value by permutation. The analysis based on pairwise distance of a multivariate data set and values were obtained using type III sums of squares with 999 permutations of residuals, considering significant difference at *p*≤0.001.

**Supplementary Table S7.** Sample type-associated relative abundances of most abundant bacterial genera (% of all reads) in feces of piglets on day 28 and day 33 of life.

| **Day of life (DoL)** | **28** | | **33** | |  | ***p-value*** | | |
| --- | --- | --- | --- | --- | --- | --- | --- | --- |
| **Sample type** | **Feces** | **Swab** | **Feces** | **Swab** | **SEM** | **DoL** | **Type** | **DoL×Type** |
| Genus | | | | | | | | |
| *Prevotella* | 3.96b | 4.25b | 6.06b | 12.04a | 0.15 | <0.001 | <0.001 | 0.002 |
| *Lactobacillus* | 7.82b | 2.07d | 12.40a | 4.29c | 0.14 | <0.001 | <0.001 | 0.753 |
| *Escherichia-Shigella* | 0.62b | 6.06a | 0.22b | 0.19b | 0.16 | <0.001 | <0.001 | <0.001 |
| *Lachnospiraceae-*1 | 2.88b | 3.07b | 4.88a | 3.88ab | 0.10 | <0.001 | 0.394 | 0.153 |
| *Treponema* | 0.85b | 2.77a | 1.00b | 2.84a | 0.15 | 0.740 | <0.001 | 0.847 |
| *Bacteroides* | 1.61b | 4.43a | 0.28c | 1.21b | 0.12 | <0.001 | <0.001 | 0.276 |
| *Campylobacter* | 0.03c | 1.60ab | 0.92b | 2.69a | 0.14 | <0.001 | <0.001 | 0.143 |
| *Alloprevotella* | 0.57b | 1.22b | 2.99a | 3.51a | 0.08 | <0.001 | 0.006 | 0.246 |
| *Rikenellaceae* RC9 gut group | 1.21b | 2.16a | 1.33b | 2.73a | 0.08 | 0.178 | <0.001 | 0.460 |
| Unclassified *Muribaculaceae* | 3.12ab | 1.50c | 3.32a | 2.34b | 0.07 | 0.012 | <0.001 | 0.071 |
| *Christensenellaceae* R 7 group | 2.48a | 2.09a | 1.65ab | 1.05b | 0.09 | <0.001 | 0.041 | 0.460 |
| *Oscillospiraceae* UCG-005 | 2.61a | 1.39b | 2.00ab | 1.45b | 0.07 | 0.235 | <0.001 | 0.136 |
| *Methanobrevibacter* | 7.05a | 0.47c | 1.75b | 0.17d | 0.08 | <0.001 | <0.001 | <0.001 |
| *Prevotellaceae* NK3B31 group | 0.89b | 0.58b | 1.72a | 2.40a | 0.07 | <0.001 | 0.695 | 0.003 |
| *Eubacterium coprostanoligenes* group | 1.61ab | 1.68a | 1.27ab | 1.13b | 0.06 | 0.002 | 0.767 | 0.420 |
| *Helicobacter* | 0.01c | 1.25a | 0.02c | 0.33b | 0.11 | 0.024 | <0.001 | 0.008 |
| *Clostridium sensu stricto-*1 | 3.53a | 1.30b | 0.53c | 0.27c | 0.07 | <0.001 | <0.001 | <0.001 |
| *Blautia* | 1.28b | 0.43c | 2.60a | 1.11b | 0.06 | <0.001 | <0.001 | 0.470 |
| *Sphaerochaeta* | 0.37bc | 1.08a | 0.27c | 0.77ab | 0.09 | 0.167 | <0.001 | 0.678 |
| *Prevotellaceae* UCG-003 | 0.19c | 0.41c | 0.85b | 2.19a | 0.06 | <0.001 | <0.001 | 0.008 |
| F082 | 0.40a | 0.69a | 0.42a | 0.36a | 0.10 | 0.294 | 0.476 | 0.221 |
| *Fusobacterium* | 0.22b | 0.91a | 0.0002b | 0.01b | 0.11 | <0.001 | 0.018 | 0.040 |
| *Ruminococcus* | 1.94a | 1.08b | 0.92b | 0.55c | 0.06 | <0.001 | <0.001 | 0.250 |
| *Coprococcus* | 1.40a | 0.51b | 1.54a | 0.74b | 0.05 | 0.063 | <0.001 | 0.421 |
| *Lachnoclostridium* | 1.16a | 1.30a | 0.33b | 0.27b | 0.06 | <0.001 | 0.997 | 0.332 |

Values are presented as least squares means ± standard error of the mean (SEM). At each time point (DoL), 192 piglets (96 piglets/replicate batch) were sampled. Weaning occurred on DoL28.

a,b,c means without a common superscript in the same row differ (*p*<0.05).

**Supplementary Table S8.** Sample color-associated relative abundances of most abundant bacterial genera (% of all reads) in feces of piglets on day 28 and day 33 of life.

| **Day of life (DoL)** | **28** | | | **33** | | |  | ***p-value*** | | |
| --- | --- | --- | --- | --- | --- | --- | --- | --- | --- | --- |
| **Sample color** | **Brown** | **Yellow** | **Swab** | **Brown** | **Yellow** | **Swab** | **SEM** | **DoL** | **Color** | **DoL ×Color** |
| Genus | | | | | | | | | | |
| *Prevotella* | 2.88c | 7.23ab | 4.27b | 7.03ab | 3.82bc | 12.07a | 0.22 | 0.006 | 0.002 | <0.001 |
| *Lactobacillus* | 5.68b | 14.41a | 2.08c | 15.65a | 5.64b | 4.32b | 0.20 | 0.149 | <0.001 | <0.001 |
| *Escherichia-Shigella* | 0.28b | 2.17ab | 6.04a | 0.08b | 0.94b | 0.18b | 0.25 | <0.001 | <0.001 | <0.001 |
| *Lachnospiraceae-*1 | 3.18b | 2.24b | 3.07b | 4.16b | 7.07a | 3.87b | 0.15 | <0.001 | 0.463 | 0.025 |
| *Treponema* | 1.19ab | 0.25ab | 2.76a | 0.82b | 1.56ab | 2.83a | 0.22 | 0.328 | <0.001 | 0.273 |
| *Bacteroides* | 1.41b | 2.22ab | 4.43a | 0.21c | 0.55bc | 1.20b | 0.18 | <0.001 | <0.001 | 0.548 |
| *Campylobacter* | 0.03b | 0.03b | 1.60ab | 0.50b | 2.68a | 2.67a | 0.21 | <0.001 | <0.001 | 0.077 |
| *Alloprevotella* | 0.56d | 0.64cd | 1.21cd | 2.04c | 6.41a | 3.48b | 0.12 | <0.001 | <0.001 | 0.002 |
| *Rikenellaceae* RC9 gut group | 1.30ab | 1.00b | 2.16ab | 1.36ab | 1.26ab | 2.73a | 0.13 | 0.334 | <0.001 | 0.729 |
| Unclassified *Muribaculaceae* | 3.29a | 2.68ab | 1.50c | 3.34a | 3.26ab | 2.34bc | 0.10 | 0.078 | <0.001 | 0.161 |
| *Christensenellaceae* R 7 group | 3.43a | 0.74c | 2.09ab | 1.76bc | 1.39bc | 1.05bc | 0.13 | 0.064 | <0.001 | 0.037 |
| *Oscillospiraceae* UCG-005 | 3.67a | 0.72b | 1.40b | 2.04b | 1.87b | 1.45b | 0.11 | 0.838 | <0.001 | <0.001 |
| *Methanobrevibacter* | 9.21a | 2.81b | 0.47c | 1.93b | 1.30bc | 0.17d | 0.12 | <0.001 | <0.001 | <0.001 |
| *Prevotellaceae* NK3B31 group | 0.92bc | 0.78bc | 0.58c | 1.87ab | 1.35abc | 2.40a | 0.10 | <0.001 | 0.511 | 0.009 |
| *Eubacterium coprostanoligenes* group | 1.46ab | 2.02a | 1.68ab | 1.23ab | 1.40ab | 1.13b | 0.08 | 0.013 | 0.409 | 0.584 |
| *Helicobacter* | 0.01b | 0.01b | 1.24a | 0.01b | 0.06b | 0.33ab | 0.16 | 0.373 | <0.001 | 0.028 |
| *Clostridium sensu stricto-*1 | 4.92a | 1.01bc | 1.31b | 0.53c | 0.51c | 0.27c | 0.11 | <0.001 | <0.001 | <0.001 |
| *Blautia* | 1.69bc | 0.52de | 0.43e | 2.44ab | 3.02a | 1.11cd | 0.08 | <0.001 | <0.001 | 0.002 |
| *Sphaerochaeta* | 0.43ab | 0.23ab | 1.08a | 0.18b | 0.58ab | 0.77a | 0.14 | 0.757 | <0.001 | 0.312 |
| *Prevotellaceae* UCG-003 | 0.22c | 0.13c | 0.41c | 0.70bc | 1.34ab | 2.18a | 0.10 | <0.001 | <0.001 | 0.006 |
| F082 | 0.60 | 0.07 | 0.69 | 0.34 | 0.67 | 0.36 | 0.15 | 0.709 | 0.604 | 0.071 |
| *Fusobacterium* | 0.005b | 2.19a | 0.91a | 0.002b | 0.01b | 0.01b | 0.16 | <0.001 | <0.001 | <0.001 |
| *Ruminococcus* | 2.38a | 1.00bc | 1.08b | 0.96bc | 0.80bc | 0.55c | 0.09 | <0.001 | <0.001 | 0.067 |
| *Coprococcus* | 1.75a | 0.70bc | 0.51c | 1.49ab | 1.69ab | 0.74c | 0.08 | 0.019 | <0.001 | 0.021 |
| *Lachnoclostridium* | 0.84ab | 2.18a | 1.30a | 0.36bc | 0.26c | 0.27bc | 0.10 | <0.001 | 0.167 | 0.021 |

Values are presented as least squares means ± standard error of the mean (SEM). At each time point (DoL), 192 piglets (96 piglets/replicate batch) were sampled.

a,b,c means without a common superscript in the same row differ (*p*<0.05).

**Supplementary Table S9.** Sample consistency-associated relative abundances of most abundant bacterial genera (% of all reads) in feces of piglets on day 28 and day 33 of life.

| **Day of life (DoL)** | **DoL28** | | | **DoL33** | | |  | ***P-value*** | | |
| --- | --- | --- | --- | --- | --- | --- | --- | --- | --- | --- |
| **Sample consistency** | **Balls** | **Liquid^1^** | **Swab** | **Balls** | **Liquid** | **Swab** | **SEM** | **DoL** | **Consistency** | **DoL × Consistency** |
| Genus | | | | | | | | | | |
| *Prevotella* | 4.01bc | 1.53c | 4.26b | 7.80ab | 3.91bc | 12.09a | 0.36 | 0.032 | 0.022 | 0.159 |
| *Lactobacillus* | 7.57b | 9.27ab | 2.09c | 18.90a | 5.18bc | 4.40c | 0.32 | 0.218 | <0.001 | 0.002 |
| *Escherichia-Shigella* | 0.46c | 28.44a | 6.04b | 0.02c | 0.90c | 0.18c | 0.40 | <0.001 | <0.001 | <0.001 |
| *Lachnospiraceae-*1 | 2.87b | 4.07ab | 3.07b | 4.13ab | 6.10a | 3.87ab | 0.25 | 0.282 | 0.701 | 0.839 |
| *Treponema* | 0.89b | 0.12b | 2.76a | 0.63b | 1.71ab | 2.82a | 0.37 | 0.545 | <0.001 | 0.680 |
| *Bacteroides* | 1.55b | 5.25a | 4.43a | 0.17c | 0.50bc | 1.20b | 0.30 | 0.002 | <0.001 | 0.695 |
| *Campylobacter* | 0.03b | 0.001b | 1.59a | 0.30b | 2.48a | 2.65a | 0.34 | 0.075 | <0.001 | 0.640 |
| *Alloprevotella* | 0.53d | 4.28ab | 1.21cd | 1.48c | 6.17a | 3.47b | 0.19 | 0.023 | <0.001 | 0.272 |
| *Rikenellaceae* RC9 gut group | 1.23b | 0.70b | 2.15ab | 1.20b | 1.55ab | 2.73a | 0.21 | 0.464 | <0.001 | 0.555 |
| Unclassified *Muribaculaceae* | 3.14ab | 1.87bc | 1.50c | 3.51a | 3.04ab | 2.34b | 0.16 | 0.200 | <0.001 | 0.388 |
| *Christensenellaceae* R 7 group | 2.52a | 1.15ab | 2.09a | 1.58ab | 1.76ab | 1.04b | 0.22 | 0.546 | 0.185 | 0.641 |
| *Oscillospiraceae* UCG-005 | 2.69a | 0.53b | 1.39b | 1.98ab | 2.03ab | 1.45b | 0.18 | 0.474 | <0.001 | 0.166 |
| *Methanobrevibacter* | 7.18a | 2.43ab | 0.47c | 2.16b | 1.22bc | 0.17d | 0.20 | 0.010 | <0.001 | <0.001 |
| *Prevotellaceae* NK3B31 group | 0.89cd | 0.66cd | 0.58d | 1.97ab | 1.38bc | 2.40a | 0.16 | 0.010 | 0.836 | 0.082 |
| *Eubacterium coprostanoligenes* group | 1.57ab | 3.61a | 1.68a | 1.09b | 1.57ab | 1.13b | 0.14 | 0.036 | 0.247 | 0.698 |
| *Helicobacter* | 0.01b | 0.002b | 1.24a | 0.01b | 0.06b | 0.33b | 0.27 | 0.778 | <0.001 | 0.077 |
| *Clostridium sensu stricto-*1 | 3.57a | 2.28ab | 1.30b | 0.51c | 0.55c | 0.27c | 0.19 | <0.001 | <0.001 | 0.005 |
| *Blautia* | 1.28b | 0.99bc | 0.43c | 2.75a | 2.40a | 1.11b | 0.14 | 0.006 | <0.001 | 0.585 |
| *Sphaerochaeta* | 0.38ab | 0.16ab | 1.08a | 0.09b | 0.73a | 0.76a | 0.22 | 0.965 | <0.001 | 0.535 |
| *Prevotellaceae* UCG-003 | 0.19c | 0.15c | 0.41c | 0.66b | 1.19b | 2.18a | 0.16 | 0.001 | <0.001 | 0.005 |
| F082 | 0.41ab | 0.18ab | 0.68a | 0.14b | 1.11a | 0.35ab | 0.24 | 0.883 | 0.198 | 0.623 |
| *Fusobacterium* | 0.21b | 0.87ab | 0.91a | 0.002b | 0.01b | 0.01b | 0.26 | 0.032 | 0.056 | 0.211 |
| *Ruminococcus* | 1.94a | 1.74ab | 1.08bc | 1.01bc | 0.79bc | 0.55c | 0.15 | 0.044 | <0.001 | 0.755 |
| *Coprococcus* | 1.40a | 1.56a | 0.51b | 1.47a | 1.66a | 0.74b | 0.14 | 0.677 | <0.001 | 0.611 |
| *Lachnoclostridium* | 1.13a | 2.28a | 1.30a | 0.36b | 0.29b | 0.27b | 0.16 | <0.001 | 0.795 | 0.420 |

Values are presented as least squares means ± standard error of the mean (SEM). At each time point (DoL), 192 piglets (96 piglets/replicate batch) were sampled.

^1^Fecal samples that were scored with 1.5 (liquid feces with minimal consistency) or 1 (entirely liquid feces) were considered as liquid samples. Balls represent feces with a score of 3.0 (pasty; moist feces with no cracks and no distinct shape) to 5.0 (hard, dry balls).

a,b,c means without a common superscript in the same row differ (*p*<0.05).

**References**

1. GfE, 2006: „Empfehlungen zur Energie- und Nährstoffversorgung von Schweinen“. Available from: https://www.dlg-verlag.de/misc/filePush.php?id=468&name=gfe_schwein.pdf
2. DLG (2008): DLG Information 1/ 2008: Empfehlungen zur Sauen und Ferkelfütterung, Herausgeber DLG-Arbeitskreis Futter und Fütterung, DLG-Verlag, Verlag Frankfurt am Main, Germany.
3. Muyzer G, de Waal,’ And EC, Uitierlinden2 AG. Profiling of Complex Microbial Populations by Denaturing Gradient Gel Electrophoresis Analysis of Polymerase Chain Reaction-Amplified Genes Coding for 16S rRNA [Internet]. APPLIED AND ENVIRONMENTAL MICROBIOLOGY. 1993. Available from: https://journals.asm.org/journal/aem
